# Supplementary material for: Empirical methods for controlling false positives and estimating confidence in ChIP-Seq peaks
Source: BMC Bioinformatics. 2008 Dec 5;9:523. doi: 10.1186/1471-2105-9-523 (PMC2628906; doi:10.1186/1471-2105-9-523)
Supplement: Additional File 1 — A variety of html documents from the USeq web site detailing the available applications, their best usage, output file type descriptions, command line menus, etc. [file 1471-2105-9-523-S1.zip › USeqUserGuides/JavaDoc.zip!/JavaDoc/trans/roc/class-use/Gr.html]

Uses of Class trans.roc.Gr


|  |  |  |
| --- | --- | --- |
| **Overview** \| **Package** \| **Class** \| **Use** \| **Tree** \| **Deprecated** \| **Index** \| **Help** | |  |
| PREV   NEXT | **FRAMES**    **NO FRAMES**     **All Classes** |  |


---


## **Uses of Class trans.roc.Gr**

| Packages that use Gr | |
| --- | --- |
| **trans.misc** | Rough working scripts. |
| **trans.roc** | For manipulating the BAC dmel spike in data and generating ROC curves. |

Uses of Gr in trans.misc

| Methods in trans.misc with parameters of type Gr | |
| --- | --- |
| `void` | `GrGraph.loadGrArray(Gr[] grs)` |
| `void` | `CalculateGeneExtensions.segmentGrArray(Gr[] grs)` |

Constructors in trans.misc with parameters of type Gr

`GrGraph(java.lang.String chromosome,
java.lang.String genomeVersion,
Gr[] grs)`

Uses of Gr in trans.roc

| Methods in trans.roc that return Gr | |
| --- | --- |
| `static Gr[]` | `Gr.parseGrGraph(GrGraph gr)` |

---


|  |  |  |
| --- | --- | --- |
| **Overview** \| **Package** \| **Class** \| **Use** \| **Tree** \| **Deprecated** \| **Index** \| **Help** | |  |
| PREV   NEXT | **FRAMES**    **NO FRAMES**     **All Classes** |  |


---
